# *LCK* over-expression drives STAT5 oncogenic signaling in *PAX5* translocated BCP-ALL patients

**DOI:** 10.18632/oncotarget.2807

**Published:** 2015-01-08

**Authors:** Valeria Cazzaniga, Cristina Bugarin, Michela Bardini, Marco Giordan, Geertruy te Kronnie, Giuseppe Basso, Andrea Biondi, Grazia Fazio, Giovanni Cazzaniga

**Affiliations:** ^1^ Centro Ricerca Tettamanti, Clinica Pediatrica, Università di Milano-Bicocca, Ospedale San Gerardo/Fondazione MBBM, Monza 20900, Italy; ^2^ Laboratory of Oncohematology, Department of Women's and Children's Health, University of Padova, Padova 35128, Italy

**Keywords:** PAX5, ETV6, LCK, STAT5, BCP-ALL

## Abstract

The *PAX5* gene is altered in 30% of BCP-ALL patients and *PAX5* chromosomal translocations account for 2–3% of cases. Although *PAX5* fusion genes significantly affect the transcription of *PAX5* target genes, their role in sustaining leukemia cell survival is poorly understood.

In an *in vitro* model of *PAX5/ETV6* leukemia, we demonstrated that Lck hyper-activation, and down-regulation of its negative regulator *Csk,* lead to STAT5 hyper-activation and consequently to the up-regulation of the downstream effectors, *cMyc* and *Ccnd2*. More important, cells from *PAX5* translocated patients show *LCK* up-regulation and over-activation, as well as STAT5 hyper-phosphorylation, compared to *PAX5* wt and *PAX5* deleted cases. As in BCR/ABL1 positive ALL, the hyper-activation of STAT5 pathway can represent a survival signal in *PAX5* translocated cells, alternative to the pre-BCR, which is down-regulated. The LCK inhibitor BIBF1120 selectively reverts this phenomenon both in the murine model and in leukemic primary cells. LCK inhibitor could therefore represent a suitable candidate drug to target this subgroup of ALL patients.

## INTRODUCTION

The *PAX5* gene belongs to the *PAX* gene family of transcription factors and is essential for B cell commitment [1]. It functions both as a transcriptional activator and a repressor of different target genes involved in lineage development. Furthermore, *PAX5* has been recently reported to be target of aberrancies, including mutations, deletions and translocations, in about 30% of pediatric patients affected by BCP-ALL, [2] the most frequent leukemia subset in children [3]. *PAX5* translocations occur in approximately 2–3% of patients, [4] with a variety of partner genes, such as transcription factors, kinases, structural proteins and others [2, 4, 5]. The t(9;12) is the most recurrent *PAX5* translocation and it encodes for the *PAX5/ETV6* fusion gene, [2, 5–8] which results in the juxtaposition of two transcription factors, fundamental in hematopoiesis and in B cell development [9]. The PAX5/ETV6 fusion protein retains the DNA binding domain of PAX5, while it substitutes its regulatory domains with the DNA binding, dimerization and transcription regulation domains of ETV6 [7].

We previously reported that PAX5/ETV6 is an aberrant transcription factor that localizes in the nucleus, [10] and alters the transcription profile of pre-BI cells, mainly deregulating genes involved in pre-BCR assembly and signaling [11]. Moreover, among the differentially expressed genes, a significant number has been described to be transcriptional direct targets of *Pax5*. One of the top-ranking up-regulated genes was the Src kinase *Lck,* a *Pax5* repressed target gene, which has a potential role in a wide range of hematological malignancies [12–16] and interestingly, acts as an inducer of Stat5 hyper-phosphorylation in Ba/F3 pro-B cells [17].

We and others previously contributed in describing the ability of PAX5/ETV6 and other PAX5 fusion proteins in impairing the rearrangement of the μ heavy chain and down-regulating the expression of genes involved in the signaling pathway downstream of the pre-BCR, [11] a mechanism known to be responsible not only for pre-BI cells differentiation but also for their proliferation and survival.

Therefore, the aim of the present study was to test the hypothesis that the activation of STAT5 through *LCK* up-regulation could represent a pro-survival signaling pathway activated by PAX5 fusion proteins and alternative to the pre-BCR, switched-off in this context. Primary cultures of wild type (wt) pre-BI cells stably transduced with the retroviral PAX5/ETV6 vector and patients primary cells carrying *PAX5* fusion genes were used to model this hypothesis.

## RESULTS

### *Lck* is up-regulated in PAX5/ETV6 transduced pre-BI cells

We recently reported that gene expression profiling (GEP) of PAX5/ETV6 transduced pre-BI revealed the up-regulation of the *Pax5* repressed target gene *Lck* (FC = 1.63, *p* < 0.05) [11, 18].

**Figure 1:**
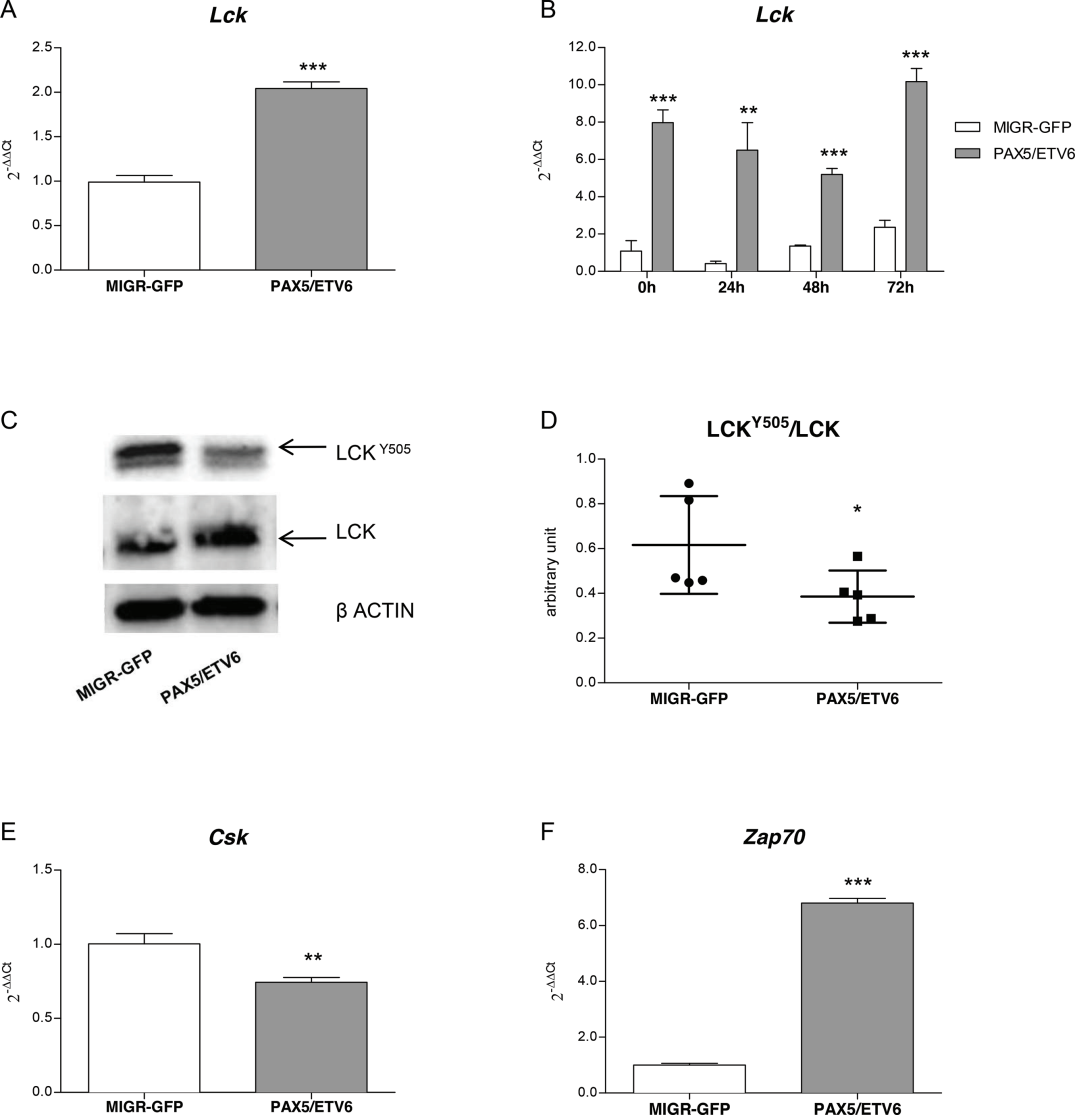
PAX5/ETV6 fusion protein leads to Lck over-activation *Lck* is up-regulated in PAX5/ETV6 transduced LY5.1FL pre-BI cells both **(A)** in basal conditions (FC = 2.04) and **(B)** in time course experiments in synchronized cells after overnight withdrawal of IL7 (FC = 7.96, FC = 15.63, FC = 3.83, FC = 4.32, at 0 h, 24 h, 48 h and 72 h, respectively). **(C)** Lck protein expression and **(D)** schematic representation of LckY505/Lck ratio, summarizing *n* = 5 western blot experiments; paired t test, *p* < 0.05. **(E)** *Csk* and **(F)** *Zap70* mRNA expression levels in LY5.1FL cells FC = 0.74 and FC = 6.8, respectively; test t, **p < 0.01; ***p < 0.001.

Quantitative PCR confirmed *Lck* over-expression at basal level in PAX5/ETV6 transduced pre-BI cells compared to the empty vector control (MIGR-GFP) (FC = 2.04, 1.55, 1.78, in LY5.1FL, B6BAFL and FLB6-67, respectively; *p* < 0.001) (Figure 1A, Supplementary Figure S1A and S2A). After synchronization by overnight withdrawal of IL7, *Lck* over-expression was confirmed at all the time points, namely 0, 24, 48 and 72 hours, with FC = 7.96 *p* < 0.001, FC = 15.93 *p* < 0.01, FC = 3.83 *p* < 0.001, FC = 4.32 *p* < 0.001 respectively, as shown in Figure 1B.

### Lck kinase activity is enhanced in PAX5/ETV6 positive cells

Lck kinase activity is regulated via the reversible phosphorylation of the negative regulatory Y505 residue in the C-terminal segment, thus inducing the clamp of the C-tail on its own SH2 domain, locking the kinase in an inactive, closed conformation [19].

The total Lck protein level was up-regulated (mean FC = 1.79, range 1.42–2.48, *p* < 0.01; mean FC = 1.97, range 1.10–3.02, *p* < 0.05; mean FC = 1.12, range 1.06–1.40, n.s., in LY5.1FL, B6BAFL and FLB6-67 cells, respectively), and PAX5/ETV6 induced a statistically significant de-phosphorylation of the Lck inhibitory domain (Lck^Y505^), causing Lck over-activation (Figure 1C-D, Supplementary Figure S1B-C, Supplementary Figure S2B-C).

Lck phosphorylation in the Y505 residue is regulated by the C-terminal Src kinase Csk, which induces a close conformation to the Lck protein, thus preventing the accessibility to its catalytic domain [19]. Indeed, we observed a significant down-regulation of *Csk* expression, which could be responsible for the de-phosphorylation at the Lck inhibitory domain (Figure 1E, Supplementary Figure S1D and S2D). Moreover the Lck target *Zap70* was found up-regulated in all the pre-BI cell populations (Figure 1F, Supplementary Figure S1E and S2E).

### *PAX5* fusion genes lead to *LCK* up-regulation in human primary cells

**Figure 2:**
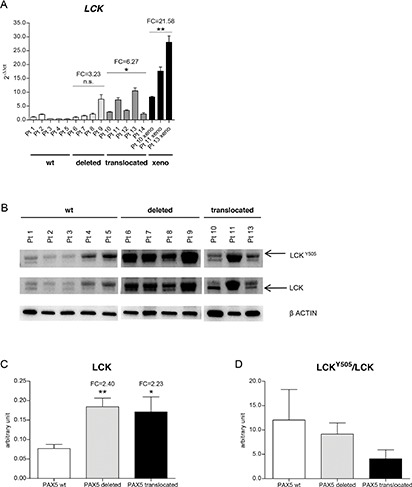
*LCK* is over-expressed in *PAX5* translocated BCP-ALL cases **(A)** *PAX5* fusion genes (dark grey) induce *LCK* over-expression in primary patients samples, compared to *PAX5* wt (white) and *PAX5* deleted BCP-ALL patients (light grey) (*PAX5* translocated cases FC = 6.27, test t, *p* < 0.05, *PAX5* deleted cases FC = 3.23, n.s.). *LCK* over-expression is maintained in *PAX5* translocated patient-derived xenograft BM cells (FC = 21.58, test t, *p* < 0.01). **(B)** Western blot analysis of LCK expression levels in *PAX5* wt, *PAX5* deleted primary patients cells and in *PAX5* translocated patient-derived xenograft BM cells. **(C)** Schematic representation of LCK expression (mean = 0.08, 0.18, 0.17 (arbitrary unit) in *PAX5* wt, *PAX5* deleted, and *PAX5* translocated cases, respectively) and **(D)** LCK^Y505^/LCK ratio, (mean = 12.03, 9.15, 4.09 in *PAX5* wt, *PAX5* deleted, and *PAX5* translocated cases, respectively). FC, fold change; xeno, *PAX5* translocated patient-derived xenografts.

In 5 BCP-ALL cases carrying *PAX5* juxtaposed to several partner genes, [20] namely *PAX5/AUTS2* (*n* = 2), *PAX5/CHFR* (*n* = 1), *PAX5/SOX5* (*n* = 1) and *PAX5/POM121C* (*n* = 1) *LCK* was found up-regulated (mean FC = 6.27, *p* < 0.05), compared to *PAX5* wt BCP-ALL patients (*n* = 5), whereas *PAX5* deleted cases showed only a higher trend of *LCK* transcript levels (Figure 2A). Patients' clinical and cytogenetics features are reported in Table 1.

**Table 1:** Clinical and cytogenetics features of analyzed patients

Patient	Gender	Age	WBC count	Immunophenotype	Karyotype	*PAX5* status (FISH)
1	F	7	44,160	cALL	nk; neg t(4;11); neg t(9;22); neg t(12;21)	wt
2	F	3	5,930	pro-B-ALL	nk; neg t(4;11); neg t(9;22); neg t(12;21)	wt
3	M	14	6,490	pre-B-ALL	nk; neg t(4;11); neg t(9;22); neg t(12;21)	wt
4	F	7	12,800	cALL	nk; neg t(4;11); neg t(9;22); neg t(12;21)	wt
5	M	8	48,700	biclonal B-ALL	nk; neg t(4;11); neg t(9;22); neg t(12;21)	wt
6	F	2	24,470	pre-B-ALL	nk; neg t(4;11); neg t(9;22); neg t(12;21)	deleted
7	F	12	75,800	cALL	nk; neg t(4;11); neg t(9;22); neg t(12;21)	deleted
8	M	14	60,700	cALL	nk; neg t(4;11); neg t(9;22); neg t(12;21)	deleted
9	M	1	81,370	pre-B-ALL	nk; neg t(4;11); neg t(9;22); neg t(12;21)	deleted
10	F	1	370,000	cALL	46, XX, der(7)t(7;11)(q11.2;q12), der(9)t(7;9)(q11.2;p13), −11, +mar[10]/46, XX[2]	Translocated *PAX5/AUTS2*
11	M	3	1,240	pre-B-ALL	45, XY, −7, der(9)t(7;9)(q22;p22)[3]/46, XY[8]	Translocated *PAX5/AUTS2*
12	M	4	19,800	cALL	46, XY, t(9;12)(p13;q24.3)[5]	Translocated *PAX5/CHFR*
13	M	10	124,000	cALL	45, XY, dic(9;12)(p13;p12.1)[6]/46, XY[3]	Translocated *PAX5/SOX5*
14	M	1	8,040	cALL	nk	Translocated *PAX5/POM121C*

WBC count, number of white blood cells/μl; M, male; F, female; nk, not known.

To better characterize the patients cohort considered for this study, we performed MLPA analysis of BM diagnostic samples (Supplementary Table S1). All the *PAX5* deleted cases presented *CDKN2A/CDKN2B* deletions (2 of them homozigous), possibly reflecting a larger deletion on chromosome 9. Patient number 9 showed *IKZF1* and *BTG1* deletions as well, and patient 7 showed *IKZF1* deletion. Therefore, at best of this knowledge, there is no evident correlation between gene deletions and different *LCK* expression levels.

Out of the 5 cases carrying *PAX5* translocations, cells were available from only 3 patients (namely, 2 PAX5/AUTS2 and PAX5/SOX5). Therefore, due to the small quantity of the samples, we expanded the cells by intravenous injection into NOD/SCID mice. When the mice became ill due to overt leukemia, they were sacrificed, and leukemia cells were harvested from the bone marrow. Leukemic infiltration was confirmed by flow cytometry, as shown in Supplementary Figure S3. Quantitative PCR confirmed that *LCK* was up-regulated in *PAX5* translocated patient-derived xenograft BM cells (mean FC = 21.58, *p* < 0.01) (Figure 2A), demonstrating that are representative of diagnostic samples.

At protein level, *PAX5* haplo-insufficiency resulted in LCK over-expression compared to wt *PAX5* status (densitometry analysis: LCK mean = 0.08, 0.18, 0.17 (arbitrary unit) in *PAX5* wt, *PAX5* deleted, and *PAX5* translocated cases, respectively) (Figure 2B-C). In addition, PAX5 fusion proteins induced marked de-phosphorylation of the LCK inhibitory domain, thus causing LCK over-activation (LCK^Y505^/LCK mean = 12.03, 9.15, 4.09 in *PAX5* wt, *PAX5* deleted, and *PAX5* translocated cases, respectively) (Figure 2B-D).

### PAX5/ETV6 induces Stat5 over-phosphorylation

We previously demonstrated in the murine model that PAX5/ETV6 cells fail to successfully rearrange the μ heavy chain and thus to express a functional pre-BCR on the cellular surface [11]. In pre-BI cells, the pre-BCR signaling is fundamental not only for their differentiation, but also for their survival and proliferation together with the IL7R pathway, [21] which leads to Stat5 phosphorylation and the consequential activation of cell cycle progression inducers. Since PAX5/ETV6 provided a survival advantage upon IL7 withdrawal, [10] we wondered if it could be due to constitutive, ligand-independent over-activation of the IL7R-Stat5 pathway. This could be exerted through *Lck* over-expression, which had been previously described to induce Stat5 hyper-phosphorylation in Ba/F3 pro-B cells [17].

**Figure 3:**
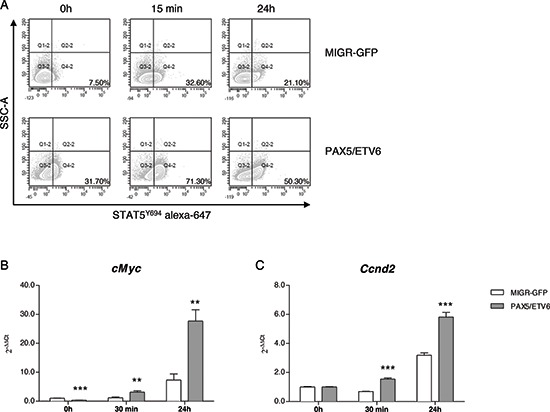
PAX5/ETV6 induces STAT5 hyper-activation in murine LY5.1FL pre-BI cells **(A)** Representative dot plots of STAT5^Y694^ FACS analysis. **(B)** RQ-PCR of *cMyc* and **(C)** *Ccnd2* at early and late time points after IL7 administration (*cMyc* FC = 0.33, FC = 2.64 and FC = 3.78; *Ccnd2* FC = 1.00, FC = 2.26 and FC = 1.83 at 0 h, 30 min and 24 h after IL7 administration, respectively). Test t, **p < 0.01; ***p < 0.001.

Indeed, phosphoflow analysis of PAX5/ETV6 transduced cells, revealed increased Stat5 phosphorylation after IL7 stimulation both at basal level and in time course experiments (Figure 3A, Supplementary Figure S4A, S5A and S6A), as confirmed by western blot (Supplementary Figure S4B, S5A and S6A). However, IL7Rα (CD127) was expressed at the same level both in PAX5/ETV6 and MIGR-GFP cells (Supplementary Figure S4C, S5B and S6B) [21].

Downstream in the pathway, we observed the up-regulation of *cMyc* and *Ccnd2*, at early and late time points after IL7 stimulation, thus indicating that Stat5 hyper-phosphorylation led to the activation of the transcription of its target genes (Figure 3B-C, Supplementary Figure S4D-E, S5C-D, S6C-D) [22].

### The Lck inhibitor BIBF1120 can revert the advantage of PAX5/ETV6 cells

**Figure 4:**
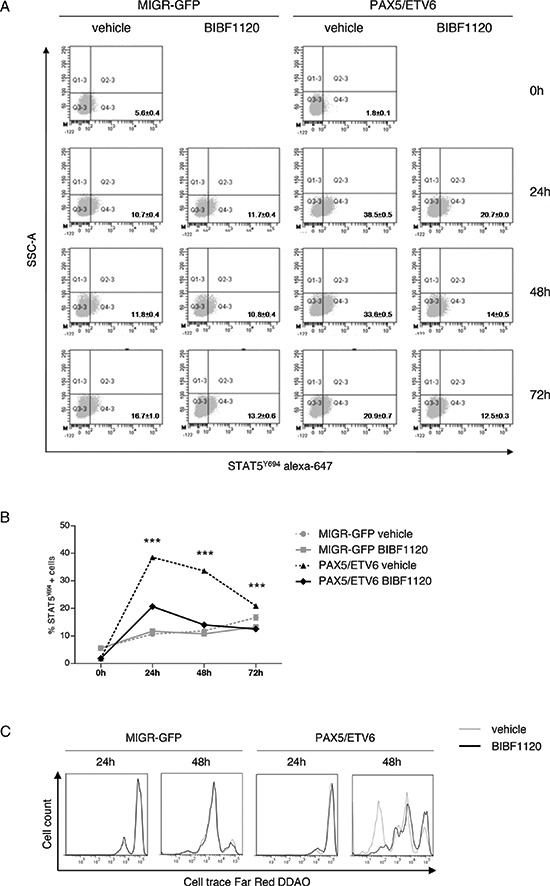
The Lck inhibitor BIBF1120 reverts the advantage of PAX5/ETV6 **(A)** Representative dot plots of STAT5Y694 FACS staining after administration of BIBF1120 in LY5.1FL cells and **(B)** relative schematic representation of the percentage of STAT5Y694 positive cells. **(C)** Increased number of replication cycles in PAX5/ETV6 transduced pre-BI cells is abrogated by BIBF1120 administration.

The treatment with the Lck inhibitor BIBF1120 [14] caused a significant reduction of Stat5^Y694^ phosphorylation in PAX5/ETV6 cells, as indicated by the decreased number of positive cells and the Mean Fluorescence Intensity (MFI), while it had no effect on MIGR-GFP control, where Lck is expressed at lower levels (Figure 4A-B). Overall, this supports the specificity of Lck activity on cell survival. By tracking of the replication cycles, we demonstrated that BIBF1120 reduces the replicative rate of PAX5/ETV6 cells, thus strikingly abrogating their proliferative advantage (Figure 4C). Indeed, cell cycle analysis showed that BIBF1120 reduced the increase in the replicative S phase exclusively in PAX5/ETV6 cells (Supplementary Figure S7A-B).

### The LCK inhibitor BIBF1120 is a novel potential candidate to target *PAX5* translocated ALL

**Figure 5:**
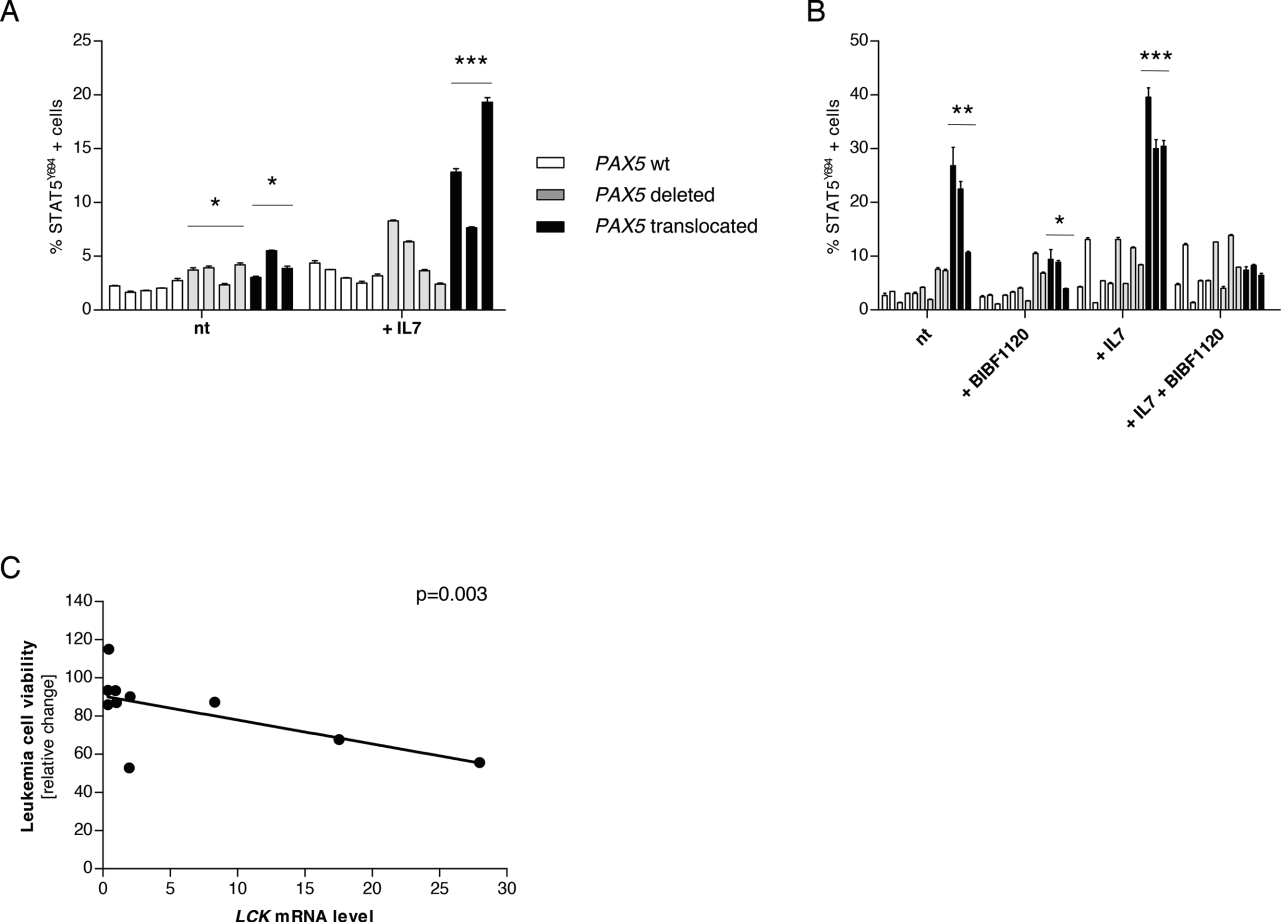
The Lck inhibitor BIBF1120 down-tunes STAT5 activation in PAX5 translocated cells **(A)** Percentage of STAT5 activation in frozen blast cells at basal level and after stimulation with hIL7 (50 ng/ml for 30 minutes). Despite the low basal level with no biological differences in the patients' groups, stimulation with hIL7 induces significantly higher STAT5 activation in *PAX5* translocated cells. **(B)** Overnight *in vitro* co-culture of blast cells on OP9 stroma confirmed that *PAX5* translocated cells show a higher STAT5 activation profile compared to *PAX5* wt and *PAX5* deleted patients. Stimulation with hIL7 increased the level of STAT5^Y694^ positive cells and *PAX5* translocated cells maintained the highest activated profile. Overnight treatment with BIBF1120 is able to reduce STAT5 activation in *PAX5* fusion gene positive cells, but it doesn't exert any significant effect on *PAX5* wt and *PAX5* deleted cells. Treatment with BIBF1120 abolishes the effect of stimulation with hIL7 in *PAX5* translocated cases, keeping the percentage of STAT5 activated cells similar to the levels of the other groups. **(C)** *LCK* levels are inversely correlated to leukemia cells viability. Test t, *p < 0.05; **p < 0.01; ***p < 0.001.

In order to verify if LCK over-expression led to STAT5 hyper-phosphorylation also in BCP-ALL cases, we perfomed phosphoflow analyses on frozen samples. Despite the low basal level of activation, *PAX5* translocated cells were more responsive to stimulation with hIL7 compared to cells from non-*PAX5* translocated patients (% STAT5^Y694^ cells mean = 3.36, 5.17, 13.27 in *PAX5* wt, *PAX5* deleted, and *PAX5* translocated cells, respectively; Figure 5A and Supplementary Figure S8).

Moreover, after co-culture of human blast cells on OP9 stroma, *PAX5* translocated cases showed higher STAT5 activation both at basal level (% STAT5^Y694^ cells mean = 2.73, 5.24, 19.98 in *PAX5* wt, *PAX5* deleted, and *PAX5* translocated cells, respectively; *p* < 0.01) and after stimulation with hIL7 (% STAT5^Y694^ cells mean = 5.81, 9.49, 33.33 in *PAX5* wt, *PAX5* deleted, and *PAX5* translocated cells, respectively; *p* < 0.001) (Figure 5B and Supplementary Figure S9). In addition, overnight treatment with BIBF1120 (50nM) reduced STAT5 phosphorylation to levels similar to *PAX5* wt and *PAX5* deleted patients (% STAT5^Y694^ cells mean = 2.46, 5.77, 7.40 in *PAX5* wt, *PAX5* deleted, and *PAX5* translocated cells, respectively; *p* < 0.05) and prevented STAT5 activation upon stimulation with hL7 (% STAT5^Y694^ cells mean = 5.81, 9.61, 7.38 in *PAX5* wt, *PAX5* deleted, and *PAX5* translocated cells, respectively; n.s.), as shown in Figure 5B and Supplementary Figure S9.

Furthermore, overnight administration of BIBF1120 was able to induce cell death in a proportional manner to *LCK* mRNA level. In particular, *LCK* levels were inversely correlated to cell viability (10 out of 12 patients with a minimal cellular viability of 15% were considered), (Figure 5C and Supplementary Figure S10). This makes BIBF1120 a candidate drug to target *PAX5* translocated ALL.

## DISCUSSION

The role of *PAX5* fusion genes in leukemogenesis and transformation events is not fully understood yet. It has been demonstrated that PAX5/ETV6, as well as other PAX5 fusion proteins, impair the expression of the μ heavy chain and down-regulates pre-BCR associated molecules at the surface level [23]. In the present study, we wanted to test the hypothesis that the activation of Stat5 through Lck up-regulation could represent a pro-survival signaling pathway activated by PAX5 fusion proteins and alternative to the non-functional pre-BCR.

Indeed, we initially showed *Lck* over-expression in an *in vitro* model of murine wt pre-BI cell populations co-coltured on OP9 stroma and transduced with PAX5/ETV6. We demonstrated that, despite a moderate over-expression of the total form of Lck protein, Lck was significantly de-phosphorylated in the inhibitory residue Lck^Y505^, thus causing Lck over-activation. Further confirming the de-phosphorylation at the Lck inhibitory domain, we demonstrated the down-regulation of the *C terminal Src kinase* (*Csk*) in PAX5/ETV6 cells. Indeed, Csk regulates Lck phosphorylation at the residue Y505, thus inducing the clamp of Lck C-tail on its own SH2 domain, locking the kinase in an inactive, closed conformation. As a confirmation of Lck hyper-activation, we demonstrated the up-regulation of its target *Zap70* [19].

Thereby, in PAX5/ETV6 cells Lck could be over-activated through two main mechanisms: a) the PAX5/ETV6 mediated up-regulation of *Lck* transcription; b) the down-regulation of *Csk*, thus resulting in the de-phosphorylation of its inhibitory domain. The same mechanism can potentially be hypothesized for other PAX5 fusion proteins.

Importantly, for the first time we showed *LCK* over-expression in bone marrow primary cells from leukemic patients carrying at diagnosis different *PAX5* fusion genes, [20] thus demonstrating that this is a common feature of *PAX5* translocations.

At protein level, not only *PAX5* translocated, but also *PAX5* deleted patients showed higher *LCK* expression compared to *PAX5* wt cases, as a result of the *PAX5* haploinsufficiency on its physiological repressed targets. However, exclusively *PAX5* fusion genes induced de-phosphorylation at the LCK^Y505^ residue, thus resulting in LCK over-activation, while *PAX*5 deleted patients didn't display strikingly over-activation of LCK. Taken together, these data suggest that *LCK* over-expression, together with its over-activation, is specifically associated to the dominant activity of PAX5 fusion proteins and contributes to the biological distinction of the *PAX5* translocated from *PAX5* deleted patients.

In murine pre-BI cells, the survival and proliferation role of the pre-BCR signaling is exerted together with the IL7R pathway, [21] which leads to Stat5 phosphorylation, and consequentially activation of cell cycle progression inducers. However, we previously demonstrated that PAX5/ETV6 cells do not express a functional pre-BCR [11] and they show a short term survival advantage upon IL7 withdrawal, which could be due to the constitutive, ligand-independent over-activation of the IL7R-Stat5 pathway. Interestingly, *Lck* over-expression had been previously described to induce Stat5 hyper-phosphorylation in Ba/F3 pro-B cells and in T lymphoma cells as well [17, 24]. This feature resembles what reported in Ph+ BCP-ALL cases, where blasts do not express a functional pre-BCR, but they show STAT5 over-activation [25–27].

Indeed, we demonstrated by phosphoflow analysis that PAX5/ETV6 transduced cells showed increased Stat5 phosphorylation, and the up-regulation of two downstream targets *cMyc* and *Ccnd2*, known to have a role in cell cycle progression and malignant transformation [22, 28, 29]. We also demonstrated that IL7Rα (CD127) was expressed at the same level both in PAX5/ETV6 and MIGR-GFP cells, thus suggesting that increased Stat5 phosphorylation was independent from classical IL7R signaling cascade [21].

Moreover, PAX5/ETV6 pre-BI cells display additional typical features of oncogenic transformation, such as an increased replicative S phase population and a faster replication rate. This phenomenon may be caused by Stat5 hyper-phosphorylation, together with the down-regulation of tumor suppressor genes (*Gadd45b* and *Lats2*) [11, 30, 31].

In the human setting, we confirmed that *LCK* over-expression leads to the over-activation of STAT5 signaling pathway both at the basal level and after stimulation with hIL7 in *PAX5* translocated cases, compared to *PAX5* wt and *PAX5* deleted blast cells.

**Figure 6:**
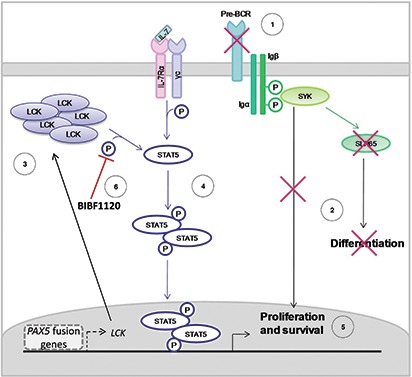
Mechanism of action of PAX5 fusion proteins in BCP-ALL The pre-BCR is not expressed in *PAX5* translocated blast cells **(A)**, causing the typical proliferation advantage and differentiation block of BCP-ALL **(B)**. In particular, PAX5 fusion proteins cause the up-regulation of LCK and its over-activation **(C)**. LCK over-activation leads to STAT5 hyper-phosphorylation **(D)**, thus sustaining proliferation and survival of blast cells via the transcription of STAT5 target genes **(E)**. The administration of the LCK inhibitor BIBF1120 reverts this phenomenon, down-tuning STAT5 signaling **(F)**.

Interestingly, STAT5 over-activation and *PAX5* haplo-insufficiency have been described as synergistic players to initiate ALL [28, 32]. In the present study we demonstrated that both these aberrancies are not only necessary to drive leukemia, but they are a direct consequence of the expression of the PAX5 fusion protein, which can a) repress the activity of endogenous PAX5 [11] and b) activate STAT5 through LCK (Figure 6).

In this scenario, PAX5/ETV6 (and PAX5 fusion proteins in general) share some features of BCR-ABL1 leukemic transformation: a) both the fusion genes impair the μ heavy chain rearrangement and the pre-BCR exposure on the cellular surface; [10, 11, 26] and b) both induce STAT5 hyper-phosphorylation, via LCK in PAX5 translocated cases and via BCR/ABL1 itself in Ph+ cells, respectively [25, 26, 32]. This similarity might at least partly explain the relatively frequent occurrence of *PAX5* abnormalities in Ph-like ALL [33].

The LCK inhibitor BIBF1120 specifically abrogated the survival advantages of both murine PAX5/ETV6 pre-BI cells and *PAX5* translocated blast cells, down-tuning STAT5 phosphorylation and preventing the effect of stimulation with IL7, indicating the specificity of the LCK direct mechanism (Figure 6).

LCK has recently been reported as a key molecule in multiple subsets of leukemia. Indeed, LCK over-activation has been found to correlate with poor response to treatment in pediatric BCP-ALL patients, [12] MLL-rearranged ALL [13] and in chronic lymphoblastic leukemia (CLL), [14, 15] thus proposing LCK activation as a putative marker for glucocorticoid resistance. Moreover, LCK has been also shown to be essential for the proliferation and survival in a subset of T-ALL characterized by NUP214/ABL1 activity (6% of T-ALL cases) [16].

Besides BIBF1120, LCK has been found as a target of several tyrosine kynase inhibitors, such as Imatinib and Dasatinib, and it has been demonstrated in multiple studies that TKI treatment specifically down-regulates *LCK* expression and diminishes LCK activation [14, 34, 35]. Therefore, the LCK driven oncogenic signaling may candidate tyrosine kinase inhibitors as a possible treatment for *PAX5* translocated patients.

## MATERIAL AND METHODS

### Ethic statement

Investigation has been conducted in accordance with the ethical standards and according to the Declaration of Helsinki and according to national and international guidelines and has been approved by the authors' institutional review board.

### Patients' cohort

*PAX5* translocated (*n* = 5), *PAX5* deleted (*n* = 4) and *PAX5* wt (*n* = 5) pediatric BCP-ALL cases enrolled in the AIEOP-BFM ALL2000 protocol and selected based on cytogenetics and FISH were considered for this study [20]. Patients' clinical and cytogenetics features are reported in Table 1.

### Multiplex Ligation dependent Probe Amplification (MLPA)

The selected cohort was screened for aberrations in B cell genes (*PAX5*, *ETV6*, *RB1*, *BTG1*, *EBF1*, *CDKN2A*, *CDKN2B*, and *P2RY8-CRLF2*) by the MLPA SALSA p335 kit (MRC Holland, Amsterdam, The Netherlands) using 125 ng of genomic DNA. The assays were performed according to the manufacturers' protocol. Electrophoresis and quantification of fluorescein amidite-labeled amp icons were performed on an ABI-3130 genetic analyzer (Applied Biosystems, Carlsbad, CA). The resulting peak intensities were normalized to the manufacturers' control probes and to normal DNA as a reference. An intensity ratio between 0.75 and 1.3 was considered to represent a normal copy number, a ratio between 0.25 and 0.75 a monoallelic deletion, and a ratio < 0.25 a biallelic deletion. The results of MLPA analysis are reported in Supplementary Table S1.

### Primary leukemia cell xenograft

BM cells (1 × 10^6^) collected at the diagnosis of *PAX5* translocated BCP-ALL patients were inoculated via intravenous injection into NOD/SCID mice (Charles River Laboratories, Wilmington, MA, USA) after sublethal irradiation (125 Rad) with Radgil (Gilardoni, Mandello (LC), Italy). When the mice became ill due to overt leukemia, they were sacrificed, and leukemia cells were harvested from bone marrow and spleen. Leukemic infiltration was confirmed by flow cytometry, as shown in Supplementary Figure 3.

### Cell cultures

Phoenix packaging cell line was cultured in DMEM high glucose, in the presence of 10% heat-inactivated fetal bovine serum (FBS; Lonza, Basel, Switzerland) at 37°C and 5% CO_2_. OP9 stroma cells were cultured in Iscove's modified Dulbecco's medium, supplemented with 5 × 10^5^ mol/L of h-mercaptoethanol, 1 mmol/L of glutamine, 0.03% w/v primatone (Sigma-Aldrich, St. Louis, Missouri, USA), 100 units/mL of penicillin, 100 Ag/mL of streptomycin, and 20% FBS at 37°C and 10% CO_2_. Wt murine pre-BI cell populations, namely LY5.1FL, B6BAFL and FLB6-67, were isolated from the fetal liver of donor mice as previously described, [10] and co-cultured on OP9 stroma in Iscove's modified Dulbecco's medium supplemented with 2% FBS, 0.03% w/v primatone, and 5% mIL7 at 37°C and 10% CO_2_ and transduced with either MIGR-PAX5/ETV6 or MIGR-GFP vector [10, 11]. Representative phenotype analyses are reported in Supplementary Figures S11–13. Patients BM cells were co-cultured on OP9 stroma in MEM alpha medium supplemented with Glutamax (Gibco, Life Technologies, Carlsbad, California, USA) and 20% FBS, at 37°C and 10% CO_2_.

### Retroviral transduction

Wt pre-BI cell populations were transduced with either MIGR-PAX5/ETV6 or MIGR-GFP vector. The retroviral supernatant was obtained using MIRUS transfection reagent (Mirus Bio LLC, Madison, Wisconsin, USA) and Phoenix packaging cell line according to the manufacturer's instruction. On day +3 post transduction, the GFP positive cells were sorted using FACS Aria sorter (Becton Dickinson Biosciences, San Jose, California, USA).

### Reverse transcription-PCR and real-time quantitative-PCR assays

RNA extraction was performed using TRIZOL reagent (Invitrogen, Life Technologies, Carlsbad, California, USA), following the manufacturer's protocol. Superscript II enzyme (Invitrogen, Life Technologies, Carlsbad, California, USA) was used for cDNA synthesis. Primers and probes for real time quantitative PCR were selected according to the Software Probe Finder (Roche Diagnostics, Basel, Switzerland). Data were expressed using the comparative ΔΔCt method. In the murine setting, the *Hprt* transcript was considered as a reference and MIGR-GFP cells as standardization control, whereas in human cell samples the *ABL1* gene was selected as a housekeeping gene, and *PAX5* wt patients as standardization controls [36]. Both t test and SD values refer to triplicates of a single experiment and 3 independent experiments were performed for each gene. Primers and probes are reported in Supplementary Table S2.

### Phosphoflow analysis

Human BM cells were stimulated with hIL7 (50 ng/ml) for 30 minutes, whereas murine pre-BI cells were stimulated with 5% mIL7 after synchronization by overnight mIL7 withdrawal. Afterwards, cells were harvested at different time points, fixed in 1.5% paraformaldehyde and permeabilized with 90% Methanol according to Nolan's Lab protocol (http://www.cytobank.org/nolanlab/experiment_protocols/general_protocol.html). Samples were stored in 90% methanol at −20°C. After recovering, they were stained by specific antibodies (Supplementary Table S3).

### Western blotting

Cells were lysed in Tris-HCl pH 7.4 20mM, NaCl 20mM, EDTA pH8 2mM, Na_3_VO_4_ 0.2mM, Triton 1%, NaF 25mM, β-glycerolphosphate 25mM, PMSF 1mM, Protease inhibitor cocktail 1x. StripAblot Stripping Buffer (Euroclone S.p.A., Pero, Italy) was used to recover membranes. Densitometry analyses were performed using Alliance instrument and Uviband software (Uvitec Cambridge, UK). Antibodies are listed in Supplementary Table S4.

### BIBF1120 treatment

Murine pre-BI cells were treated with either 50nM LCK inhibitor BIBF1120 (Selleckchem, Houston, Texas, USA) or vehicle (DMSO) after synchronization by overnight IL7 withdrawal. Human blast cells were cultured overnight with 50nM LCK inhibitor BIBF1120 or vehicle.

### Viability assay

The viability of human leukemia cells was evaluated by staining with BD Fixable Viability Stain V450, according to manufacturer's protocol, and analyzed by BD FacsCanto II (Becton Dickinson Biosciences, San Jose, California, USA).

### Proliferation assay

Cells were stained for 15 minutes with 1μM CellTrace^TM^ Far Red DDAO-SE (Life Technologies, Carlsbad, California, USA), washed twice with PBS-20% FBS and then analyzed with BD FacsCanto II (Becton Dickinson Biosciences, San Jose, California, USA).

### Cell cycle analysis

Pre-BI cells were fixed in PFA 1%, permeabilized with 70% ethanol and stained with PI solution (40 μg/ml PI supplemented with RNase). Samples were acquired with BD FacsCalibur (Becton Dickinson Biosciences, San Jose, California, USA) and analyzed with FlowJo software.

## SUPPLEMENTARY FIGURES AND TABLES



## Abbreviations

BCP-ALL: B cell precursors acute lymphoblastic leukemia
wt: wild type
BCR: B cell receptor
IL7: interleukin 7
IL7R: IL7 receptor
