# Supplementary material for: LCK over-expression drives STAT5 oncogenic signaling in PAX5 translocated BCP-ALL patients
Source: Oncotarget. 2015 Jan 8;6(3):1569–81. doi: 10.18632/oncotarget.2807 (PMC4359315; doi:10.18632/oncotarget.2807)
Supplement: Supplementary file 1 [file oncotarget-06-1569-s001.pdf]

SUPPLEMENTARY FIGURES AND TABLES

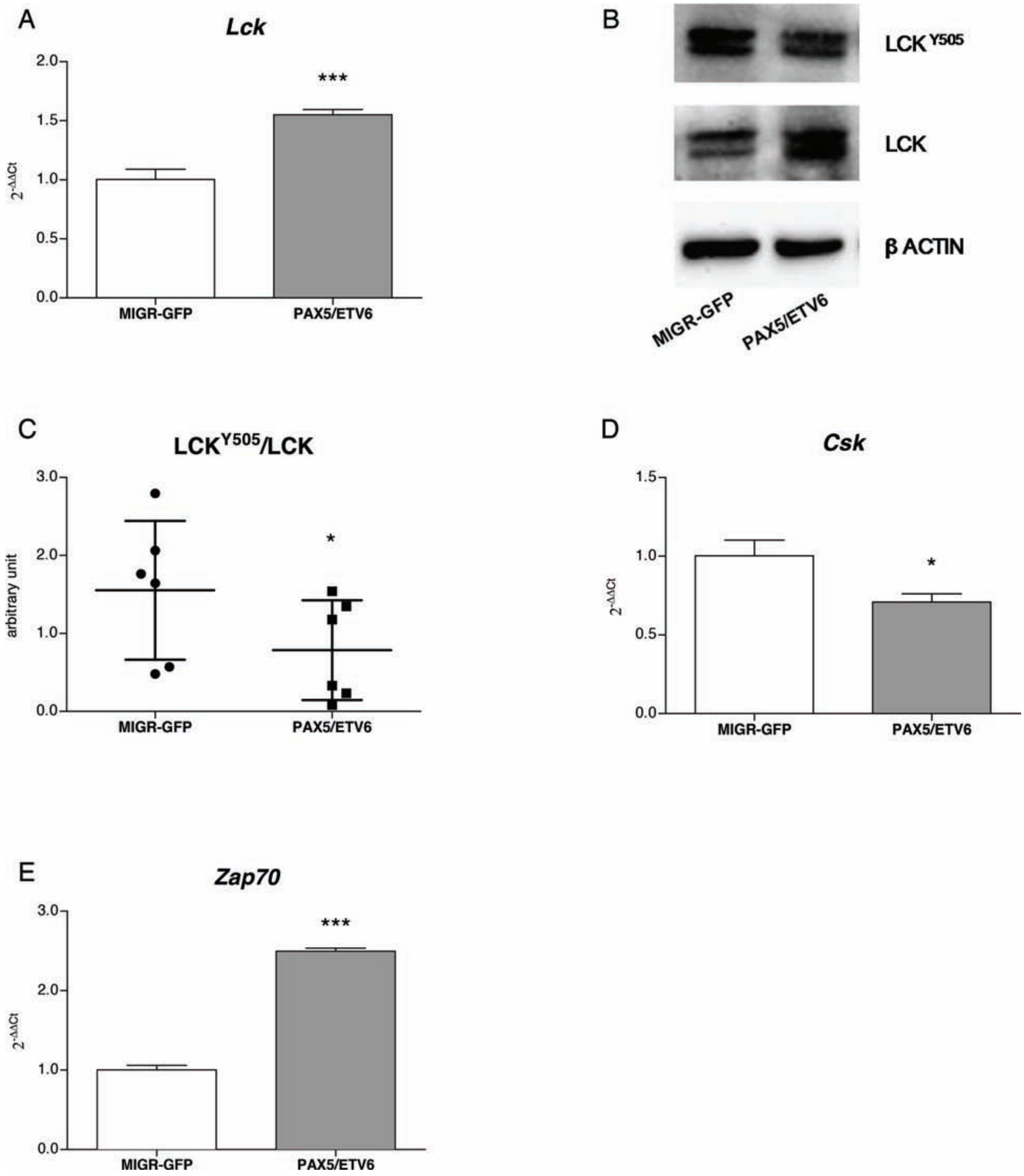

Supplementary Figure S1: (A) *Lck* mRNA expression levels in PAX5/ETV6 transduced B6BAFL pre-BI cells (FC = 1.55). **(B)** Lck protein expression and **(C)** schematic representation of LckY505/Lck ratio in B6BAFL pre-BI cells, summarizing $n = 6$ western blot experiments. **(D)** *Csk* and **(E)** *Zap70* mRNA expression levels determined by RQ-PCR (FC = 0.71 and FC = 2.50, respectively). Test t, $*p < 0.05$; $***p < 0.001$.

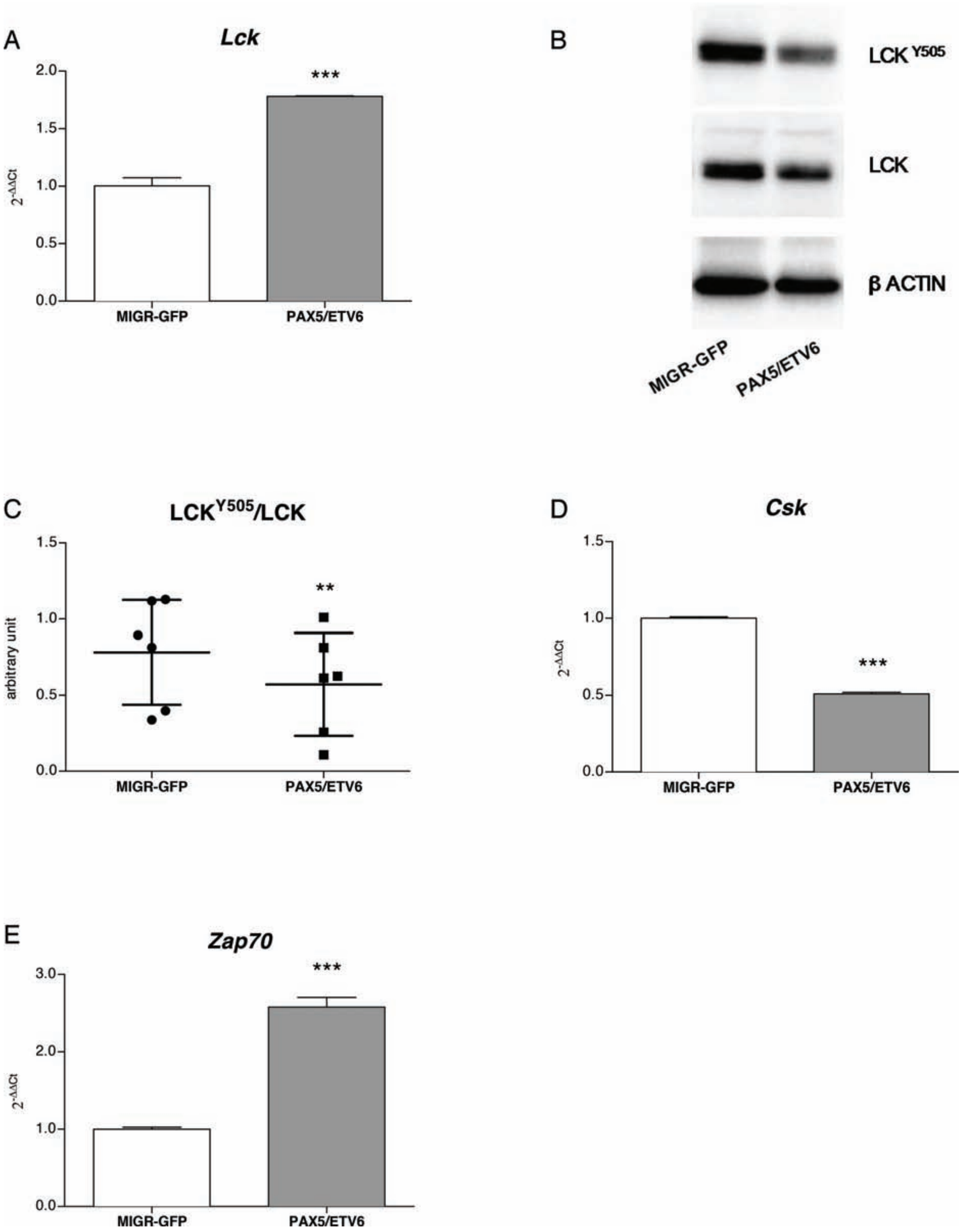

Supplementary Figure S2: **(A)** *Lck* mRNA expression levels in PAX5/ETV6 transduced FLB6-67 pre-BI cells (FC = 1.78). **(B)** LCK protein expression and **(C)** schematic representation of LckY505/Lck ratio in FLB6-67 pre-BI cells, summarizing $n = 6$ western blot experiments. **(D)** *Csk* and **(E)** *Zap70* mRNA expression levels determined by RQ- PCR (FC = 0.51 and FC = 2.58, respectively). Test t, $**p < 0.01$; $***p < 0.001$.

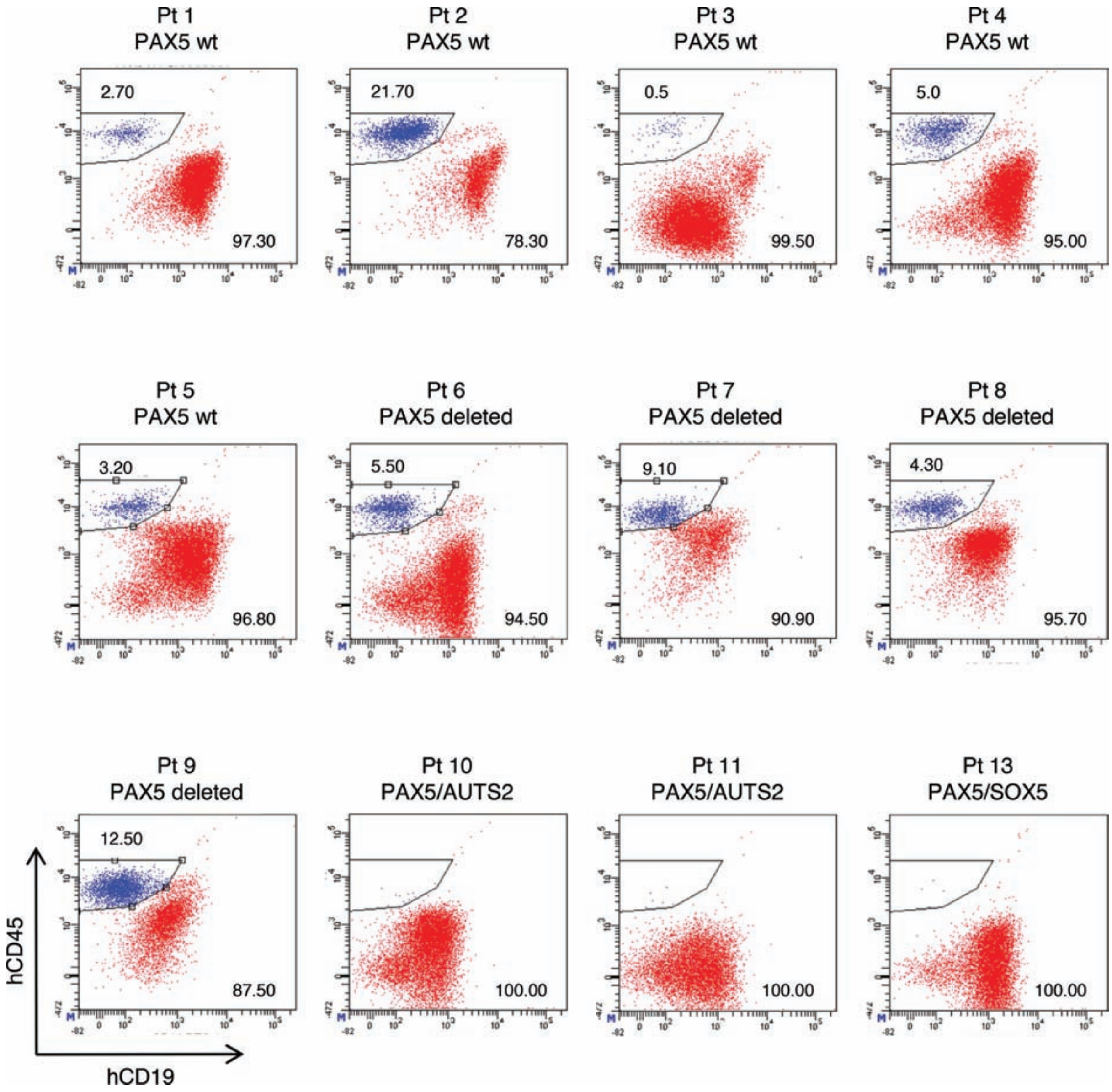

Supplementary Figure S3: Phenotypic analysis of BM blast cells of the selected cohort. Red population, blasts ($CD10^{+}$ and $CD45^{low}$); blue population, normal residue ($CD45^{high}$). Gate on viable cells, $CD10^{+}$.

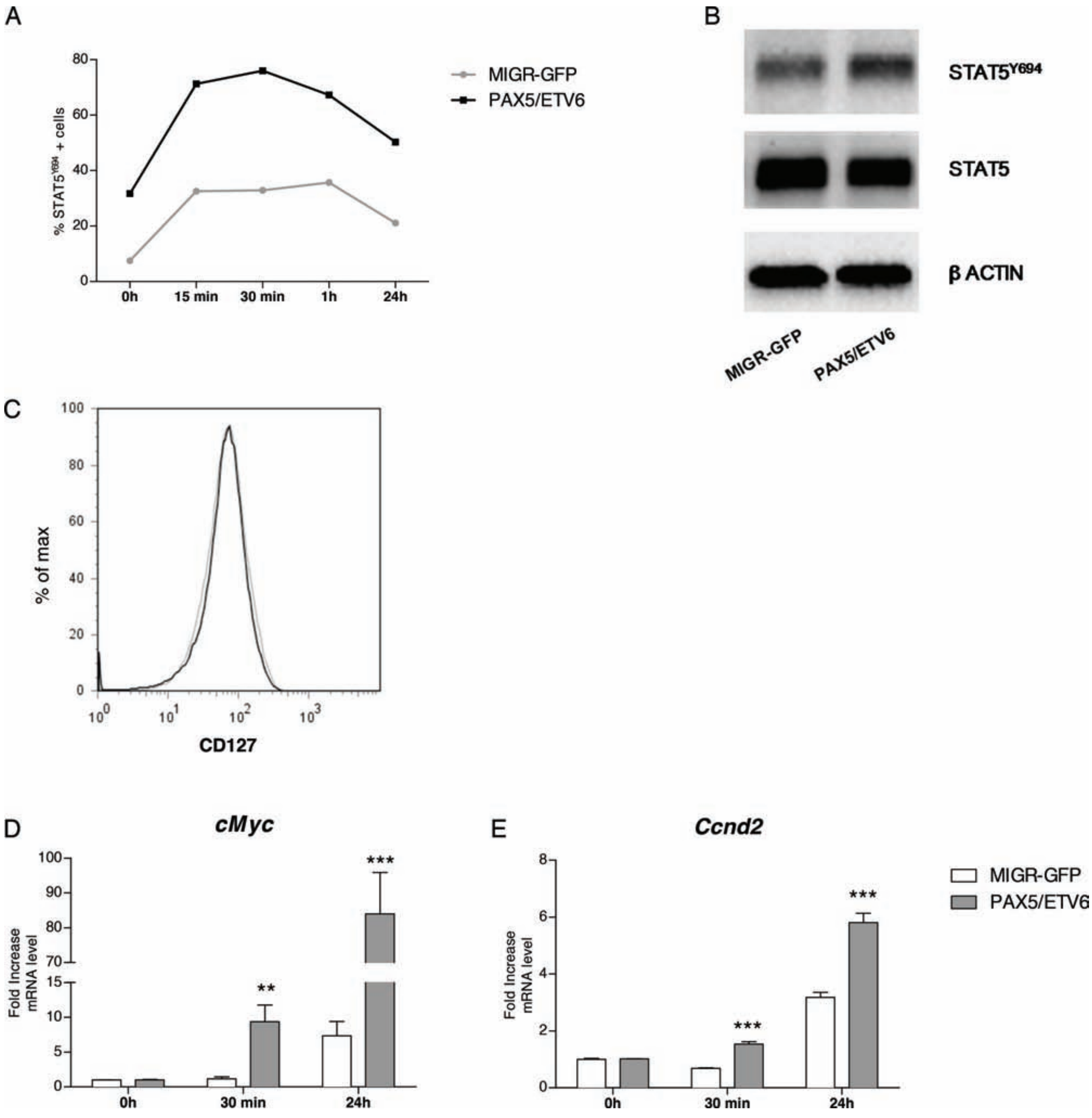

Supplementary Figure S4: (A) Schematic representation of the percentage of $STAT5^{Y694}$ positive cells in time course experiment in LY5.1FL pre-BI cells. **(B)** Western blot analysis showing the increased expression of $STAT5^{Y694}$ in PAX5/ETV6 cells (FC = 1.40), while the total form expression levels are comparable in both cell populations. **(C)** CD127 protein expression is similar in PAX5/ETV6 (black line) versus MIGR-GFP transduced cells (grey line). **(D)** Fold increase calculated on time 0 h of *cMyc* mRNA expression levels (PAX5/ETV6 FI = 9.37 and FI = 83.97; MIGR-GFP FI = 1.18 and FI = 7.32 at early and late time points, respectively). **(E)** Fold increase calculated on time 0 h of *Ccnd2* mRNA expression levels (PAX5/ETV6 FI = 1.54 and FI = 5.81; MIGR-GFP FI = 1.18 and FI = 3.18 at early and late time points, respectively). Test t, $**p < 0.01$; $***p < 0.001$.

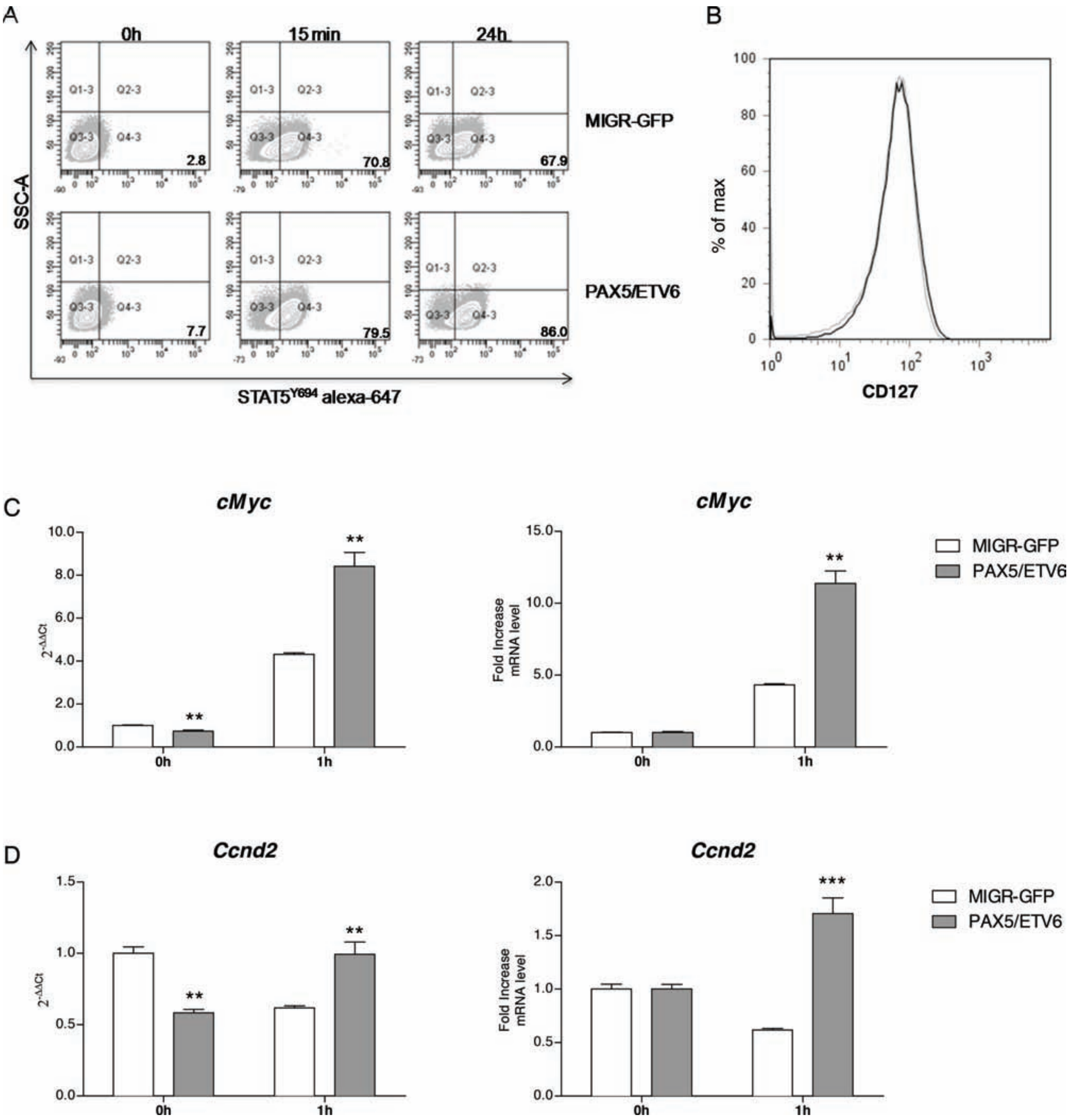

Supplementary Figure S5: (A) Representative dot plots of STAT5^{Y694} phenotypes in B6BAFL cells. **(B)** CD127 protein expression is similar in PAX5/ETV6 (black line) versus MIGR-GFP transduced cells (grey line). **(C)** RQ- PCR of *cMyc* transcript expression (FC = 0.74 and FC = 1.95 at 0 h and 1 h, respectively) and correspondent fold increase calculated on time point 0 h after IL7 administration (PAX5/ ETV6 FI = 11.36 and MIGR-GFP FI = 4.32, at 1 h). **(D)** *Ccnd2* expression levels (FC = 0.58 and FC = 1.60 at 0 h and 1 h, respectively) and correspondent fold increase calculated on time 0 h after IL7 administration (PAX5/ETV6 FI = 1.71 and MIGR-GFP FI = 0.62, at 1 h). Test t, $^{**}p < 0.01$; $^{***}p < 0.001$.

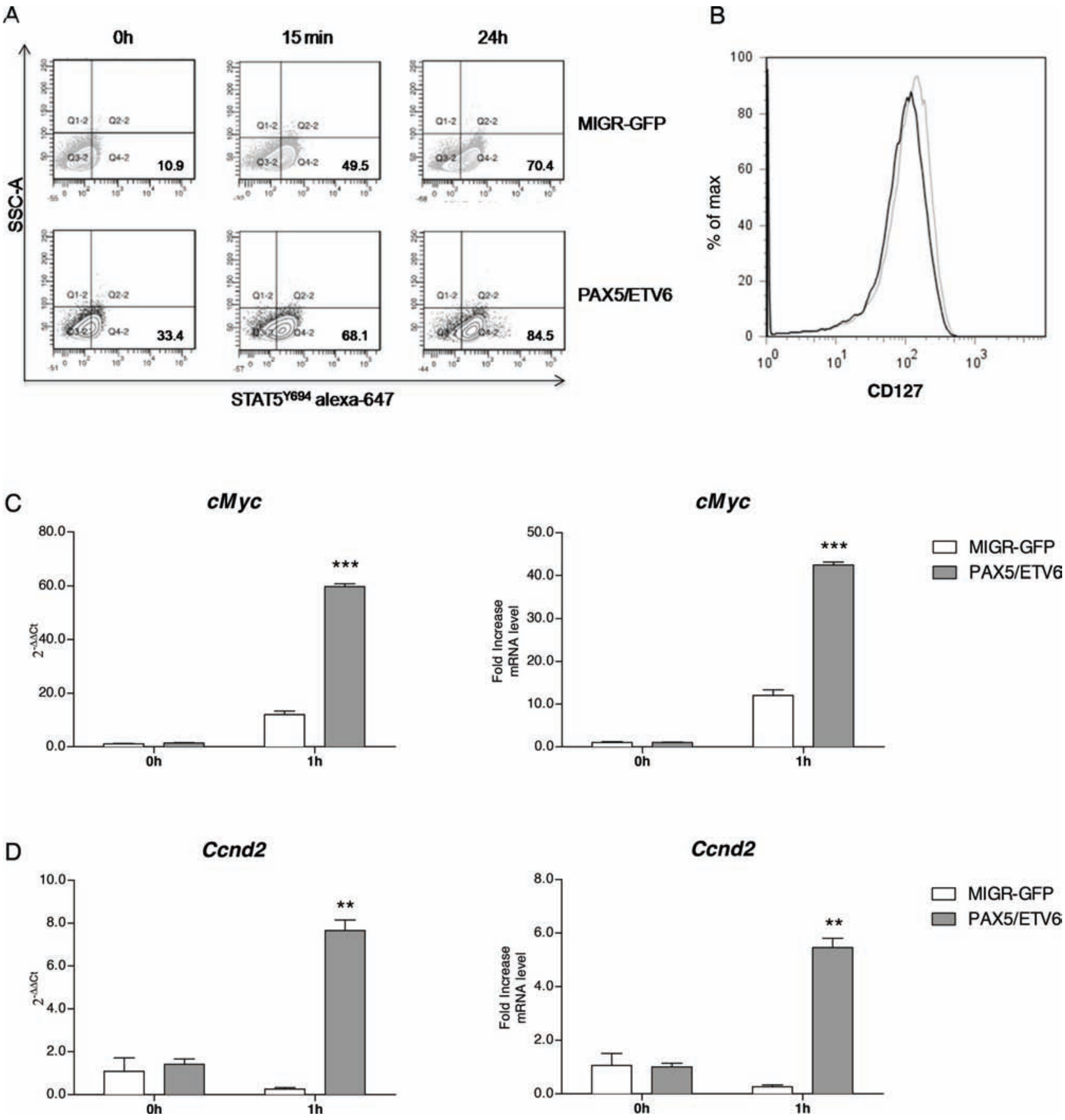

Supplementary Figure S6: **(A)** Representative dot plots of STAT5^{Y694} phenotypes in FLB6-67 cells. **(B)** CD127 protein expression is similar in PAX5/ETV6 (black line) versus MIGR-GFP transduced cells (grey line). **(C)** RQ- PCR of cMyc transcript expression (FC = 1.41 and FC = 4.96 at 0 h and 1 h, respectively) and correspondent fold increase calculated on time point 0 h after IL7 administration (PAX5/ETV6 FI = 42.42 and MIGR-GFP FI = 12.06, at 1 h). **(D)** *Ccnd2* expression levels (FC = 1.41 and FC = 28.37 at 0 h and 1 h, respectively) and correspondent fold increase calculated on time 0 h after IL7 administration (PAX5/ETV6 FI = 5.45 and MIGR-GFP FI = 0.27, at 1 h). Test t, $^{**}p < 0.01$; $^{***}p < 0.001$.

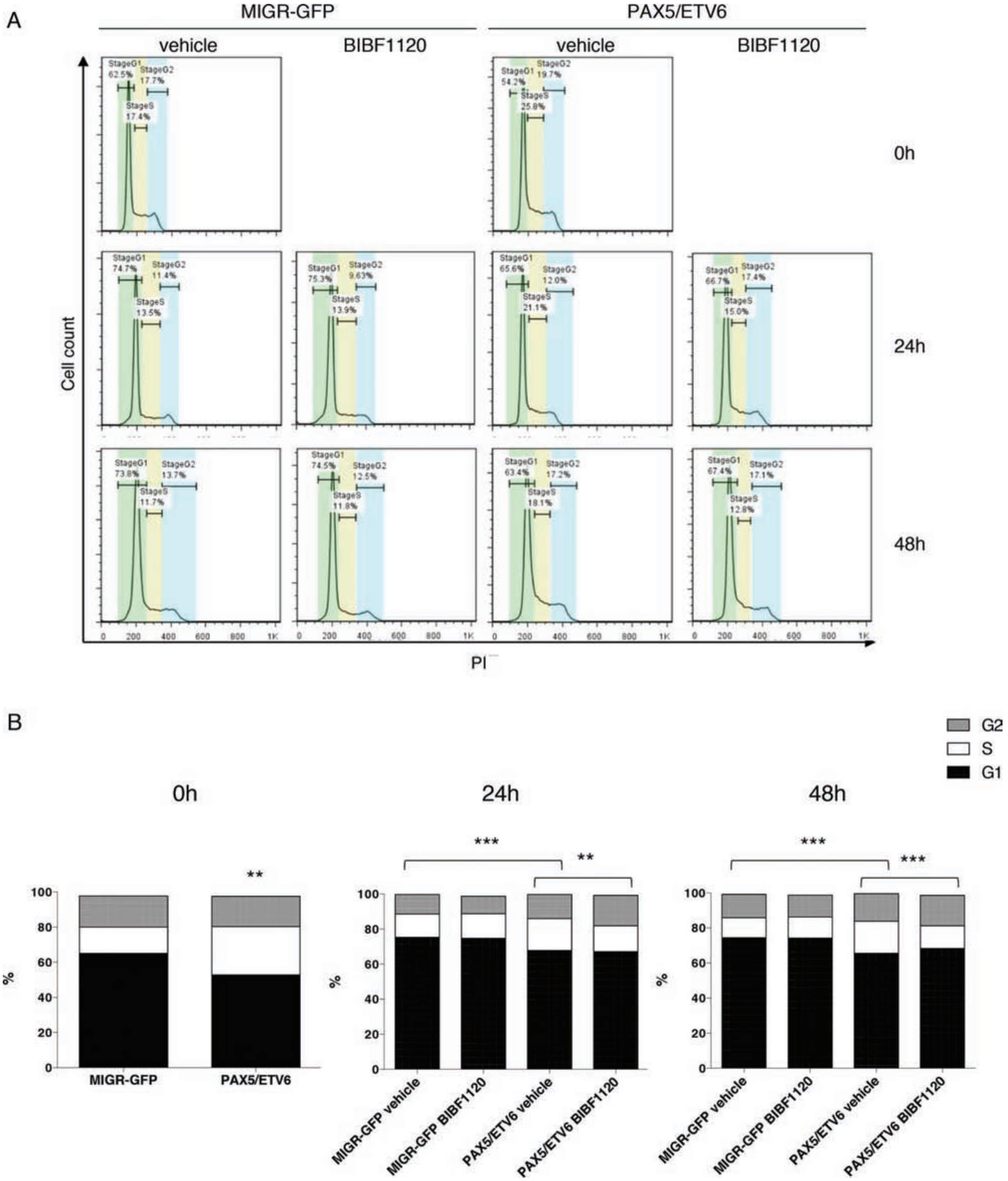

Supplementary Figure S7: (A) Representative cell cycle analysis in LY5.1FL cells. G1 phase, green; S phase, yellow; G2 phase, blue and **(B)** relative evaluation. Test t, $**p < 0.01$; $***p < 0.001$.

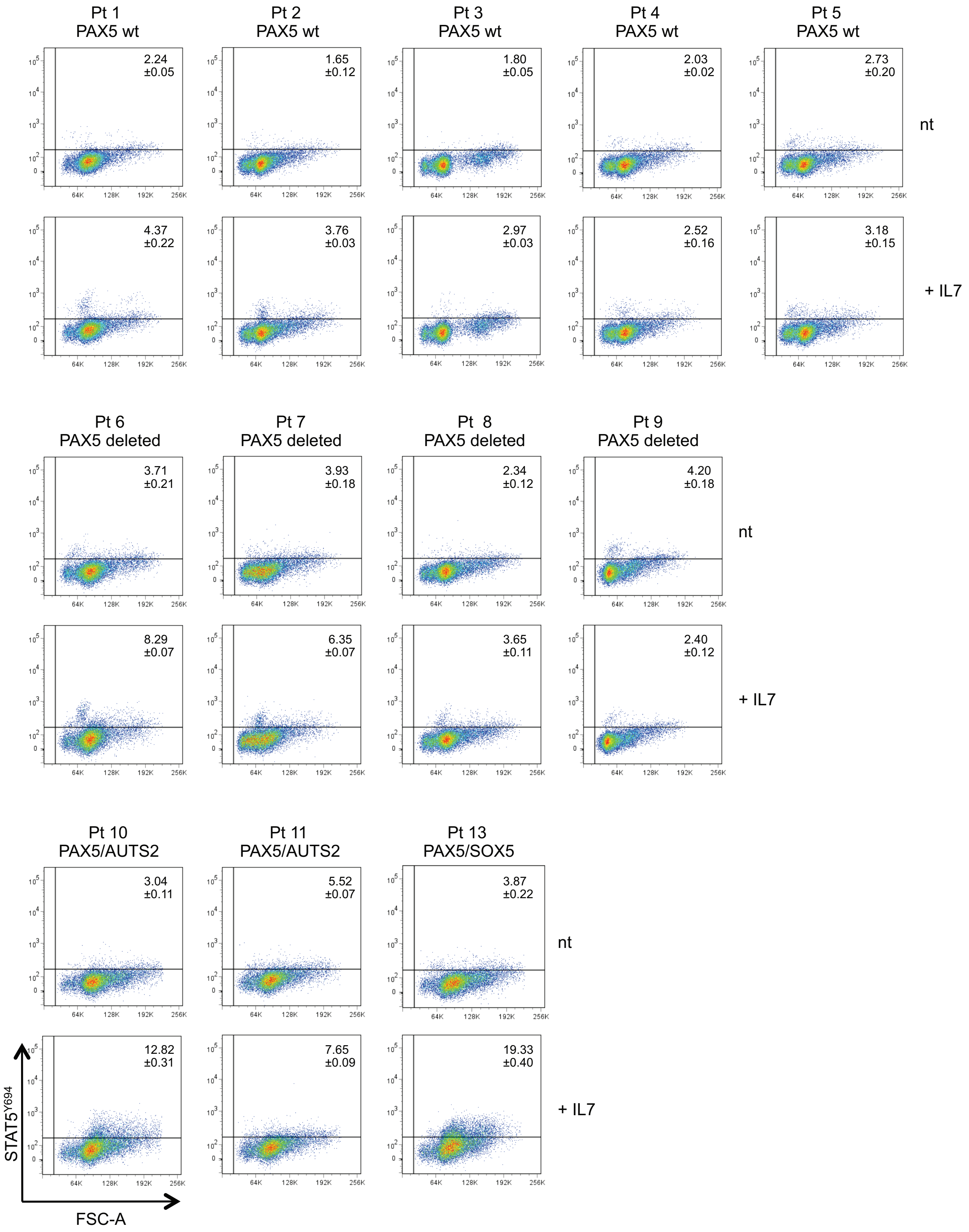

Supplementary Figure S8: Representative dot plots of STAT5^{Y694} FACS staining after thawing and stimulation with 50ng/ml hIL7. Gate on viable lymphocytes, CD10^{+} and CD45^{low}.

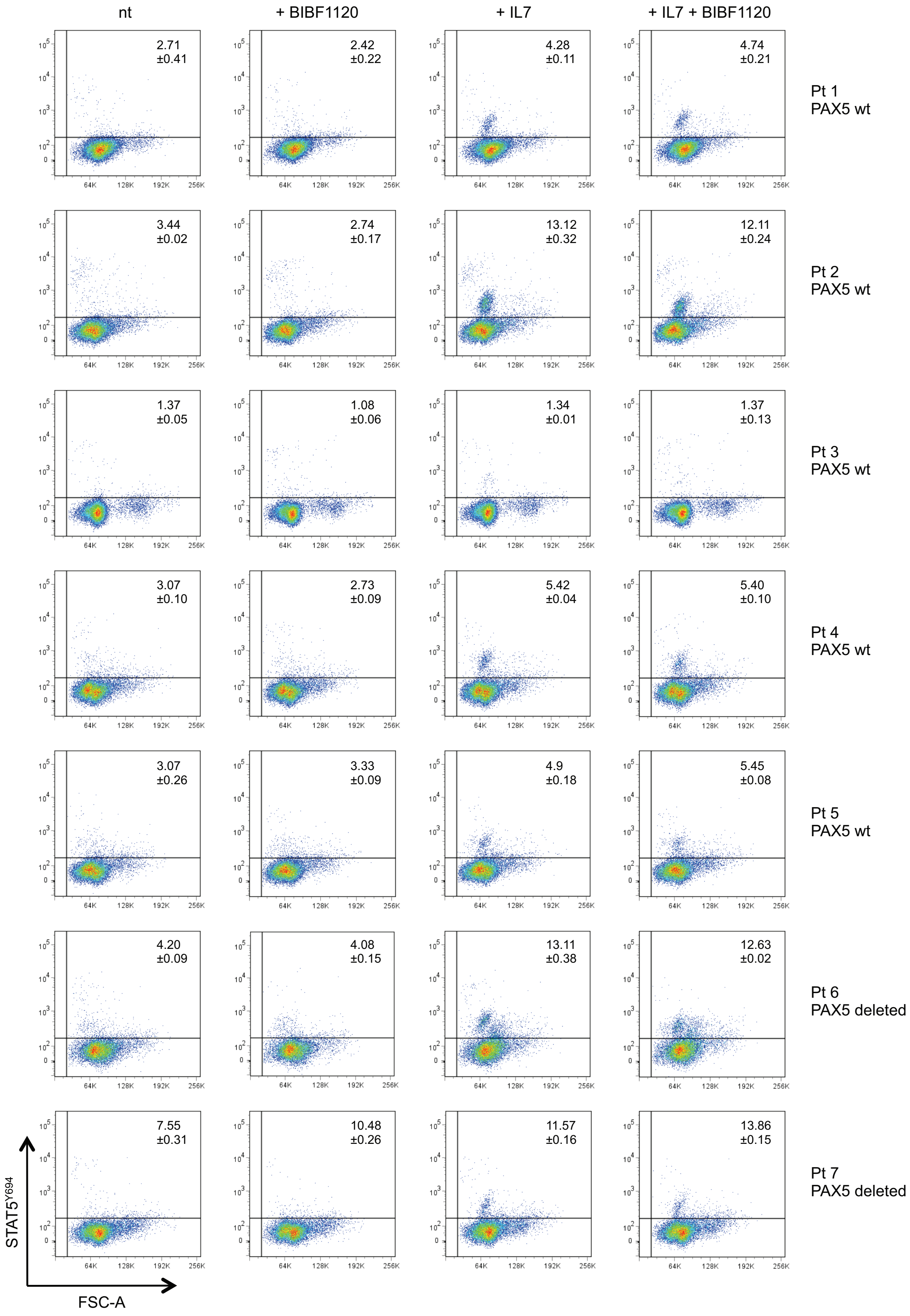
nt
+ BIBF1120
+ IL7
+ IL7 + BIBF1120
2.71 ±0.41
2.42 ±0.22
4.28 ±0.11
4.74 ±0.21
Pt 1 PAX5 wt
3.44 ±0.02
2.74 ±0.17
13.12 ±0.32
12.11 ±0.24
Pt 2 PAX5 wt
1.37 ±0.05
1.08 ±0.06
1.34 ±0.01
1.37 ±0.13
Pt 3 PAX5 wt
3.07 ±0.10
2.73 ±0.09
5.42 ±0.04
5.40 ±0.10
Pt 4 PAX5 wt
3.07 ±0.26
3.33 ±0.09
4.9 ±0.18
5.45 ±0.08
Pt 5 PAX5 wt
4.20 ±0.09
4.08 ±0.15
13.11 ±0.38
12.63 ±0.02
Pt 6 PAX5 deleted
7.55 ±0.31
10.48 ±0.26
11.57 ±0.16
13.86 ±0.15
Pt 7 PAX5 deleted
STAT5Y694
FSC-A

(*Continued*)

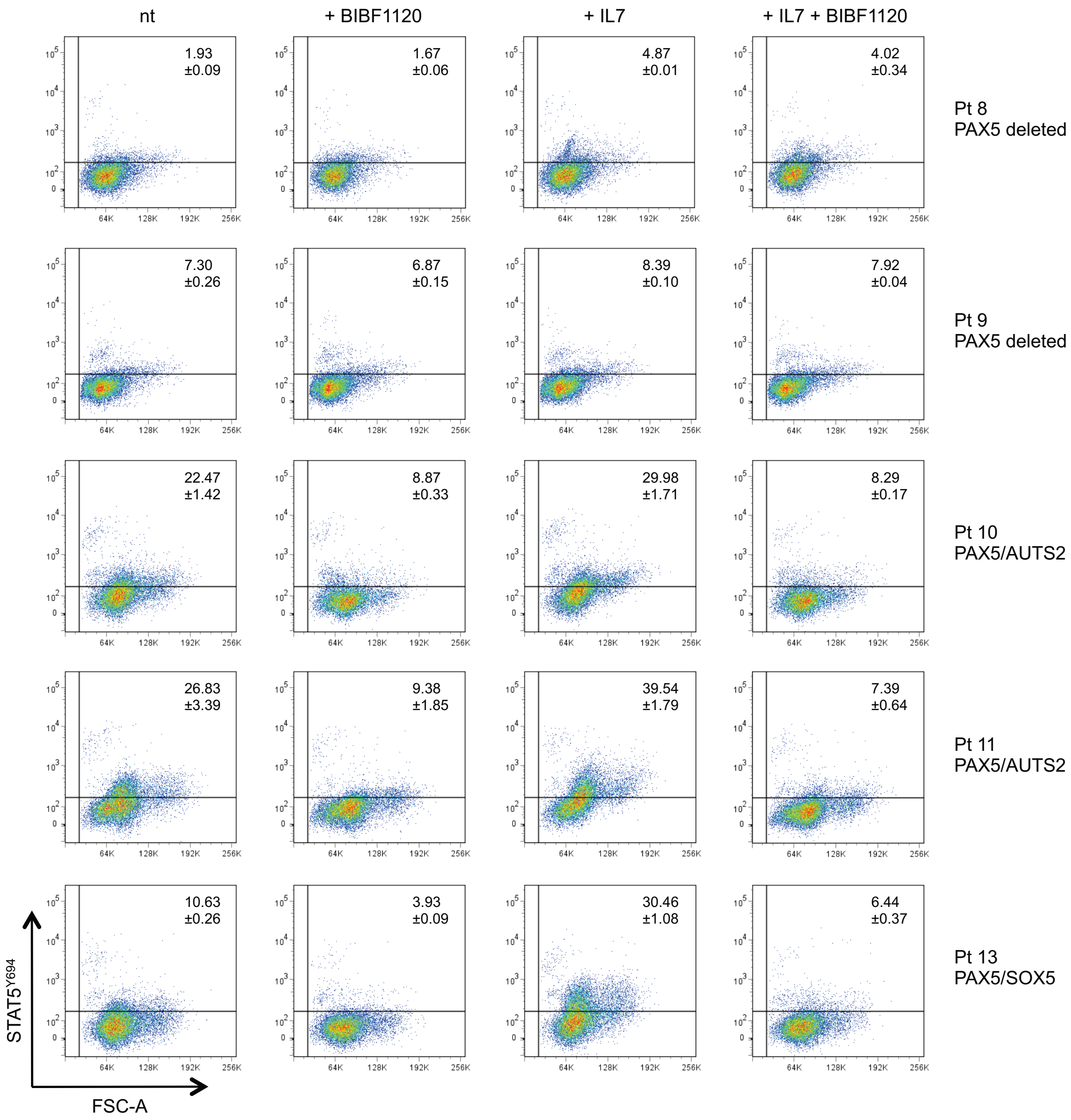

Supplementary Figure S9: Representative dot plots of STAT5^{Y694} FACS staining after overnight treatment of BIBF1120 and stimulation with or without 50ng/ml hIL7. Gate on viable lymphocytes, hCD10^{+} and hCD45^{low}.

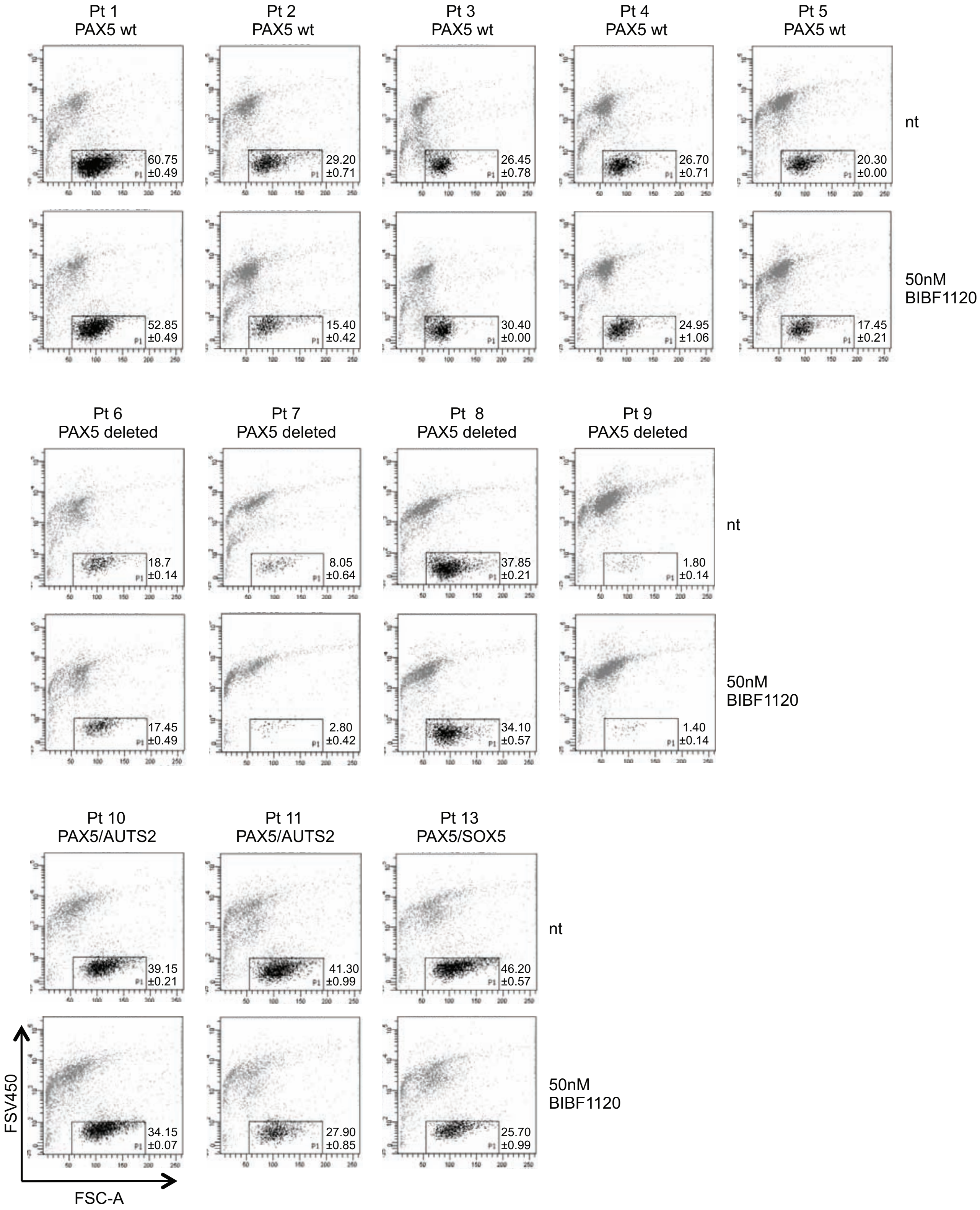

Supplementary Figure S10: Representative dot plots of BD Horizon Fixable Viability staining after overnight treatment with either BIBF1120 or vehicle.

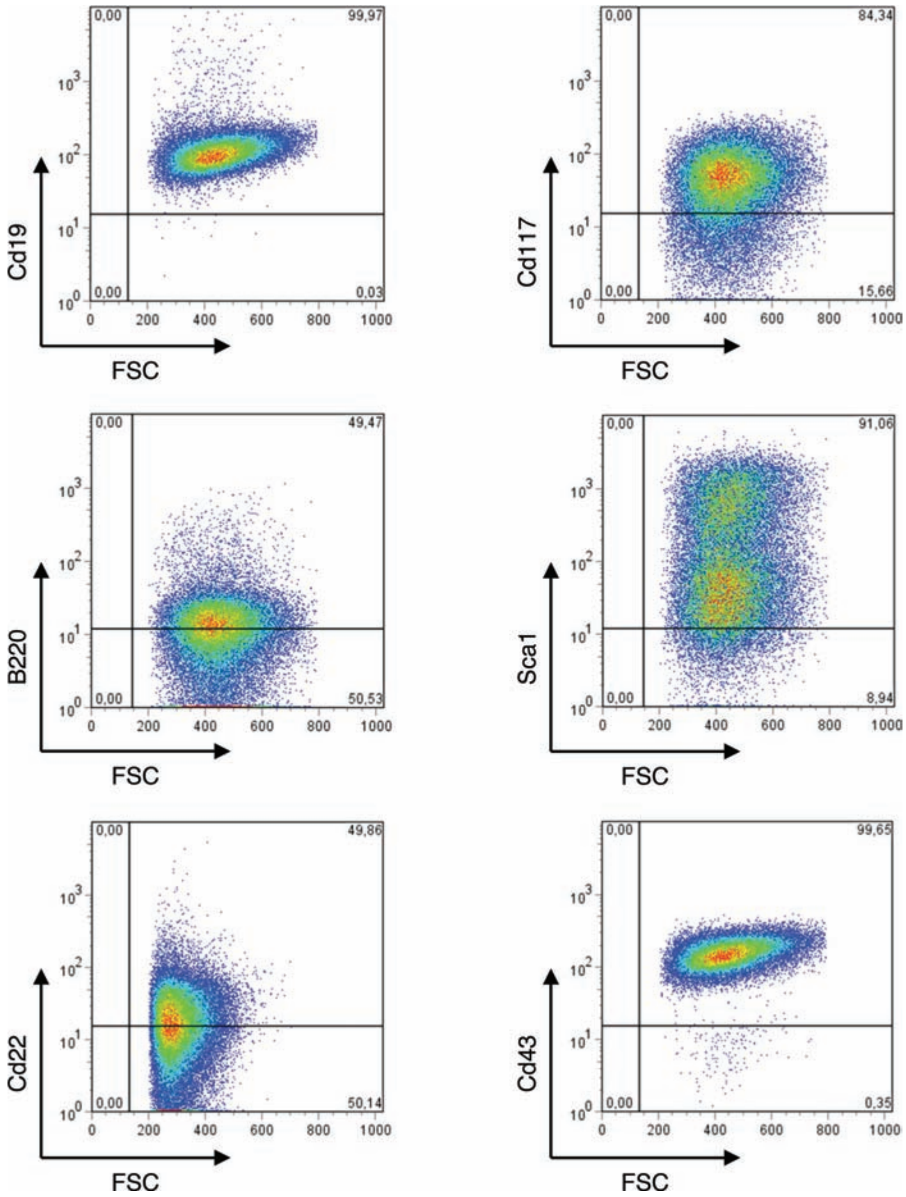

Supplementary Figure S11: Representative phenotypic characterization of LY5.1FL cells.

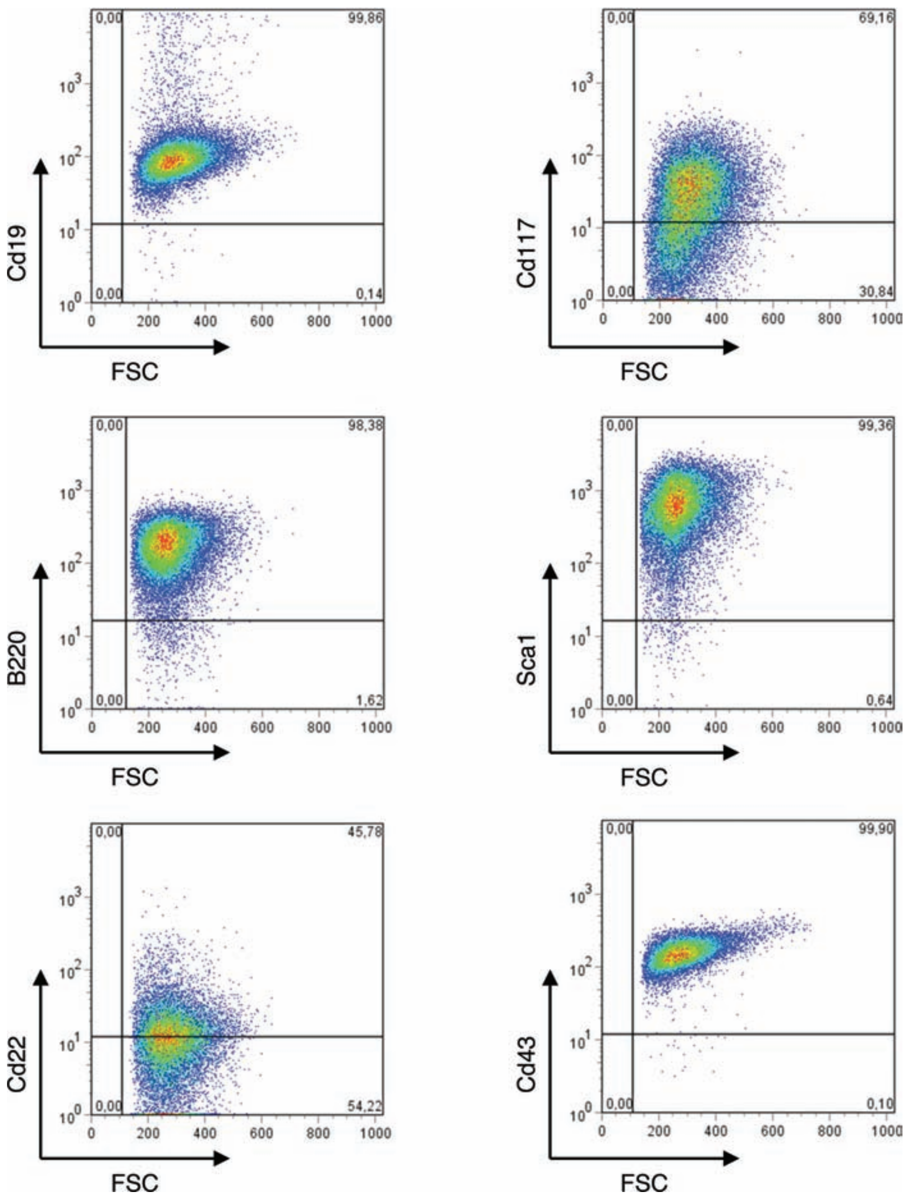

Supplementary Figure S12: Representative phenotypic characterization of B6BAFL cells.

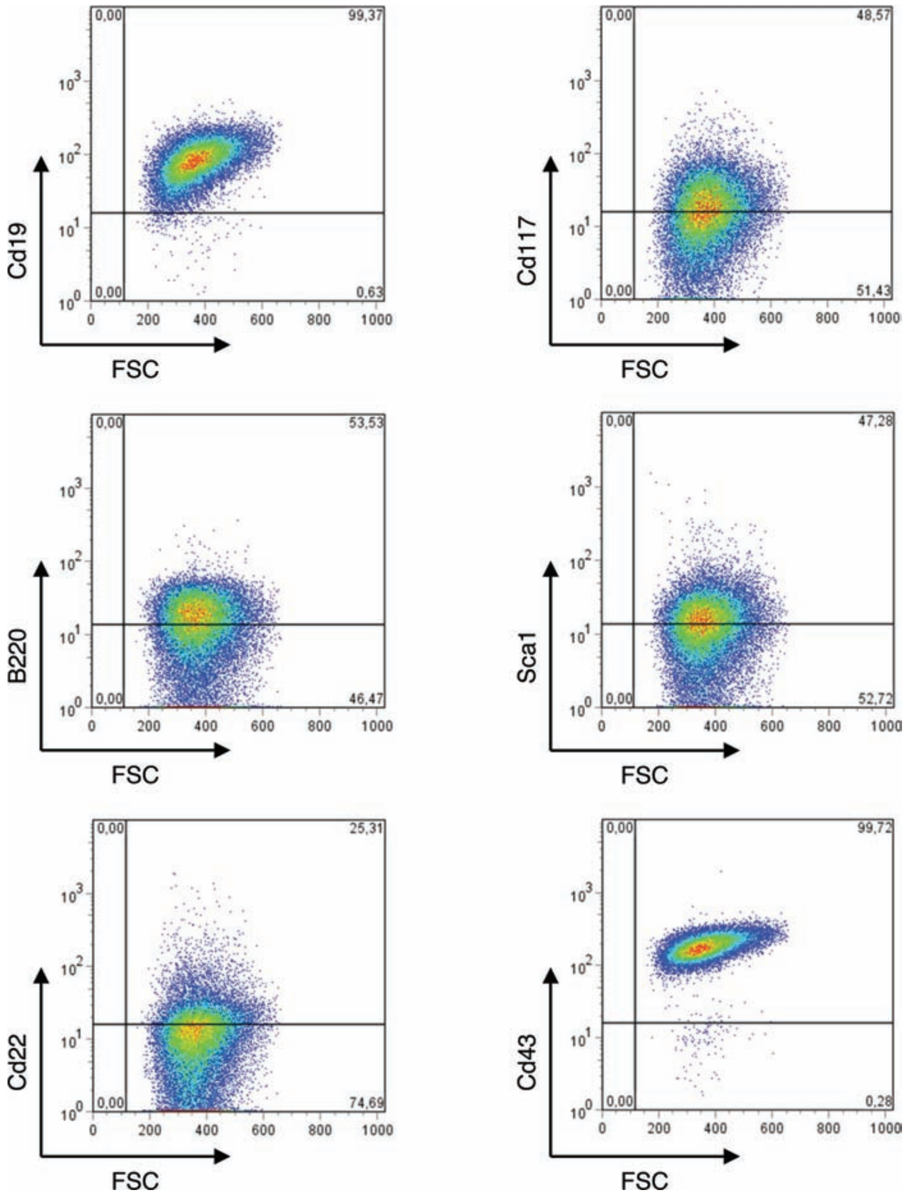

Supplementary Figure S13: Representative phenotypic characterization of FLB6-67 cells.

Supplementary Table S1. MLPA analysis of selected patients

Patient	*PAX5*	*P2RY8-CRLF2*	Chr X	*EBF1*	*IKZF1*	*CDKN2A*	*CDKN2B*	*ETV6*	*BTG1*	*RB1*
1	wt	wt	wt	wt	deleted	wt	wt	wt	wt	wt
2	wt	wt	wt	wt	wt	gain	gain	wt	wt	wt
3	wt	wt	wt	wt	wt	wt	wt	wt	wt	wt
4	wt	wt	wt	wt	deleted	wt	wt	wt	wt	wt
5	wt	wt	wt	wt	wt	wt	wt	wt	wt	wt
6	deleted	wt	gain	wt	wt	deleted	deleted	wt	wt	wt
7	deleted	wt	wt	wt	deleted	deleted*	deleted*	wt	wt	wt
8	deleted	wt	wt	wt	wt	deleted	deleted	wt	wt	deleted
9	deleted	wt	wt	wt	deleted	deleted*	deleted*	wt	deleted	wt
10	Translocated P*AX5/AUTS2*	wt	wt	wt	wt	deleted*	deleted*	wt	wt	gain
11	Translocated *PAX5/AUTS2*	wt	wt	wt	wt	wt	wt	wt	wt	wt
12	Translocated *PAX5/CHFR*	wt	wt	wt	deleted	deleted	deleted	wt	wt	wt
13	Translocated *PAX5/SOX5*	wt	wt	wt	wt	deleted*	deleted*	deleted	deleted	wt
14	Translocated *PAX5/POM121C*	wt	wt	wt	wt	deleted*	deleted*	wt	wt	wt

*homozigous.

Supplementary Table S2. List of primers and UPL probe numbers used

Gene name	Primer sequence	Probe number
h_*ABL1* L	aggaatccagtatctcagacgaa	57
h_*ABL1* R	ggaggtcctcgtcttggtg	
h_*LCK* L	agtcagatgtgtggtcttttgg	18
h_*LCK* R	cctccgggttggtcatc	
m_*Ccnd2* L	ctgtgcatttacaccgacaac	45
m_*Ccnd2* R	cactaccagttcccactccag	
m_*cMyc* L	cctagtgctgcatgaggaga	77
m_*cMyc* R	tccacagacaccacatcaattt	
m_*Csk* L	gcctgaagccttgagagaga	31
m_*Csk* R	ttcaggggaattcttgggta	
m_*Hprt* L	ggagcggtagcacctcct	69
m_*Hprt* R	ctggttcatcatcgctaatcac	
m_*Lck* L	cgtgtgtgaaaactgccact	21
m_*Lck* R	gagatccctcataggtgaccag	
m_*Zap70* L	cagaccgacggcaagttc	12
m_*Zap70* R	ccatagaccagggacagtgc	

Supplementary Table S3. Antibodies used for FACS analysis

Antigen	Fluorochrome	Source
Human CD10	PE	eBioscience
Human CD19	FITC	BD biosciences
Human CD45	PerCP	BD biosciences
Human/mouse STAT5^{Y694}	Alexa-488	BD biosciences
Human/mouse STAT5^{Y694}	Alexa-647	BD biosciences
Mouse B220	APC	eBioscience
Mouse Cd19	PE	eBioscience
Mouse Cd22	PE	eBioscience
Mouse Cd43	PE	eBioscience
Mouse Cd117	PE	eBioscience
Mouse Sca1	APC	eBioscience

Supplementary Table S4. List of antibodies used for western blotting

Antigen	Dilution	Source
LCK^{Y505}	1:1000	Cell Signaling Technology
LCK	1:1000	Cell Signaling Technology
$STAT5^{Y694/Y699}$	1:1000	Merck-Millipore
STAT5 (3H7)	1:1000	Cell Signaling Technology
ACTB	1:2000	Sigma-Aldrich
